# Progression of mild traumatic brain injury with intracranial hemorrhage: a retrospective study of associated factors in patients managed conservatively in the emergency department

**DOI:** 10.1186/s12245-026-01290-2

**Published:** 2026-07-11

**Authors:** Supapitch Chingthongkham, Welawat Tienpratarn, Pawarisa Thongpuntair, Supitcha Chingthongkham, Sirote Wongwaisayawan

**Affiliations:** 1 Faculty of Medicine, Ramathibodi Hospital, Mahidol University, Bangkok, 10400 Thailand; 2 Department of Emergency Medicine, Faculty of Medicine, Ramathibodi Hospital, Mahidol University, 270 Rama VI Road, Thung Phaya Thai, Ratchathewi, Bangkok, 10400 Thailand; 3 Emergency Radiology Unit, Department of Diagnostic and Therapeutic Radiology, Faculty of Medicine, Ramathibodi Hospital, Mahidol University, Bangkok, 10400 Thailand

**Keywords:** Brain injuries, Traumatic, Intracranial hemorrhages, Disease progression, Risk factors, Tomography, X-Ray Computed, Emergency service, Hospital

## Abstract

**Background and objective:**

Patients with mild traumatic brain injury (mTBI) presenting with intracranial hemorrhage (ICH) represent a heterogeneous group, with some experiencing clinical or radiologic deterioration despite initial stability. This study aimed to identify factors associated with clinically significant progression, defined as clinical or radiological worsening resulting in hospital admission or neurosurgical intervention, among patients with mTBI and ICH evaluated in the Emergency Department (ED) of Ramathibodi Hospital, Thailand.

**Methods:**

A retrospective review was conducted of adult patients with mTBI (Glasgow Coma Scale [GCS] 13–15) and radiologically confirmed ICH who presented to the ED between 2019 and 2023. Data were retrieved from the hospital electronic medical record system. Patients managed conservatively with at least one repeated computed tomography (CT) scan were included in the study. Clinical and radiologic variables were analyzed using multivariable logistic regression to identify independent factors associated with progression.

**Results:**

Among 114 eligible patients, 26 (22.8%) demonstrated progression requiring hospital admission or neurosurgical intervention. Independent predictors of progression were midline shift (OR 11.58, 95% CI 2.93–45.78, p < 0.001), systolic blood pressure ≥160 mmHg (OR 5.49, 95% CI 1.69–17.77, p = 0.005), multiple ICH (OR 3.69, 95% CI 1.13–11.98, p = 0.030), and initial GCS 13–14 (OR 4.86, 95% CI 1.18–20.08, p = 0.029).

**Conclusion:**

These findings may help guide decisions regarding closer observation and repeat CT brain imaging in patients at higher risk of progression.

## Introduction

Mild traumatic brain injury (mTBI) is a common condition encountered in the emergency department (ED). When accompanied by intracranial hemorrhage (ICH), however, management becomes more complex, as some patients may subsequently develop clinical or radiologic deterioration despite initial stability. Accordingly, careful assessment, close observation, and repeated computed tomography (CT) imaging are often required to detect potential progression. Identification of risk factors associated with progression is therefore crucial for guiding the management and assessing the need for hospital admission. Risk stratification may help clinicians provide intensified monitoring for high-risk patients while avoiding unnecessary hospitalization or repeat CT imaging in lower-risk patients [1]. Previous studies have identified several factors associated with adverse outcomes and progression in mTBI. Patient characteristics such as sex and age appear to influence outcomes. Female patients show a higher rate of poor outcomes [1]. Although mortality rates are similar between sexes, in contrast, male patients have been reported to have longer intensive care unit length of stay (ICU-LOS), higher craniotomy rates, and higher incidence of ventilator-associated pneumonia (VAP) [2]. Increasing age is associated with worse outcomes [1, 3], and age greater than 65 years is considered an indication for cranial CT [4] and a risk factor for ICH progression [5].

Medication use also influences outcomes. While some studies have shown no significant difference in the rate of mTBI-related ICH between users and non-users of antithrombotic agents [6], others have demonstrated an increased risk of hemorrhage and progression in antiplatelet [7] and antithrombotic therapy [5], especially in patients with subdural hemorrhage (SDH) [8]. In addition, underlying diseases of patients may also worsen progression. For example, hypertension has been implicated in exacerbating cerebrovascular oxidative stress following mTBI in animal models experiment [9].

Initial CT findings are also significantly associated with progression. Features such as skull fractures [3], extradural hemorrhage (EDH), cerebral contusions, SDH thickness greater than 5 mm, and the presence of multiple ICH lesions have all been associated with an increased risk of ICH progression [5]. Moreover, SDH radiological patterns have been linked to greater neurological deterioration and a higher likelihood of requiring surgery than other patterns [8]. These risk factors can be used to determine appropriate management, including 24-hour observation, hospital admission, and discharge planning. Low-risk patients may not require in-hospital observation and can be safely discharged [10–13]. However, prior studies have not comprehensively identified factors that can reliably distinguish high-risk from low-risk patients.

Therefore, this study aimed to identify factors associated with clinically significant progression, defined as clinical or radiological worsening resulting in hospital admission or neurosurgical intervention, among patients with mTBI and ICH presenting to the ED.

## Materials and methods

### Study design and setting

This retrospective study was conducted in the ED, Faculty of Medicine Ramathibodi Hospital, Thailand. Ramathibodi Hospital is a tertiary-care university hospital and one of the largest referral centers in Thailand. It provides comprehensive medical services such as ED and trauma center, intensive care units, operating rooms, and advanced laboratory facilities. There are six main buildings which serve more than 5,000 outpatient visits per day with a capacity of over 1,000 inpatient beds across multiple specialized departments and units. For the emergency department, there is an administrative office of the emergency medicine unit, an emergency room, and 2 observation units. Observation unit 1 accommodates 23 beds for female patients and 13 beds for pediatric patients, while observation unit 2 provides 23 beds for male patients. Besides clinical services, it is also an academic center for medical, residency, and nursing students. Furthermore, it has been the data center for research works along with creating innovative medical treatments.

### Participants

This study investigated adult patients aged 18 years and older who presented to the ED with a history of head injury. CT brain imaging and repeat CT imaging were performed according to physician assessment and institutional practice, particularly in patients with neurological changes or concern for progression. The inclusion criteria were: Glasgow Coma Scale (GCS) of 13–15; radiologically confirmed ICH on initial CT brain; initial conservative management in the observation unit of ED; and at least one repeat CT brain within 24 h. Patients were excluded if they had concomitant injuries involving body regions other than the head, were pregnant, were referred to another hospital, or had incomplete data, including the absence of CT brain results.

### Data gathering and variable measurement

Data were collected between 2019 and 2023. Demographic and clinical variables, including age, sex, initial vital signs, GCS, mechanism of injury, underlying diseases, antithrombotic medication use, were collected from Ramathibodi electronic medical records. Radiological variables, including type of intracranial hemorrhage, presence of multiple ICH, and midline shift on initial CT brain imaging, were obtained from CT brain reports and the Picture Archiving and Communication System (PACS). Collected data were recorded using Microsoft Excel and analyzed to identify factors associated with progression in patients with mTBI and ICH managed conservatively in the ED.

### Outcome measurement

The primary outcome of this study was to identify factors associated with clinical or radiological progression of intracranial hemorrhage in patients with mTBI managed conservatively in the ED. Clinical progression was defined as neurological deterioration documented in the medical records, while radiological progression was defined as worsening ICH on repeat CT brain imaging as documented in radiological reports. Progression was considered clinically significant when it resulted in hospital admission or neurosurgical intervention.

### Sample size estimation

Because this study was a retrospective cohort study, all consecutive eligible patients presenting during the study period were included. Therefore, a formal a priori sample size calculation was not performed. The final sample size was determined by the number of adult patients with mTBI and radiologically confirmed ICH who met the eligibility criteria and underwent repeat CT brain imaging between 2019 and 2023.

### Statistical analysis

Baseline prognostic factors were presented as a table. Descriptive statistics were used for categorical data, which were summarized as frequencies and percentages (%), while continuous data were presented as mean and standard deviation (SD) for normally distributed variables, and as median and interquartile range (IQR) for non-normally distributed variables. All variables were initially explored to examine differences in their distribution between the progression and non-progression groups. Continuous variables were compared using means and t-test/Rank sum test, while categorical variables were compared using proportions and exact probability tests. Univariable and multivariable logistic regression analyses were performed to identify factors associated with progression in patients with mTBI and intracranial hemorrhage managed conservatively in the ED using Stata version 17.0 (StataCorp LLC, College Station, TX, USA). Results were reported as odds ratios (ORs), 95% confidence intervals (CIs), and p-values. The discriminatory ability of individual variables included in the final multivariable model, as well as the overall multivariable model, was additionally assessed using receiver operating characteristic (ROC) curve analysis and reported as the area under the ROC curve (AUC). Statistical significance was defined as a two-tailed p-value < 0.05. Given the limited number of outcome events, only a limited number of clinically relevant variables were included in the multivariable model to reduce the risk of overfitting.

### Ethics approval

This study was approved by the Human Research Ethics Committee, Faculty of Medicine, Ramathibodi Hospital, Mahidol University (COA No. MURA2025/13), approved on January 6, 2025. The requirement for informed consent was waived due to the study’s retrospective design and the use of anonymized data.

## Result

A total of 3,571 adult patients with mild traumatic brain injury (mTBI) who underwent initial CT brain imaging were identified during the study period. Of these, 3,253 patients had no evidence of ICH, while 318 patients had ICH on initial CT.

**Fig. 1:**
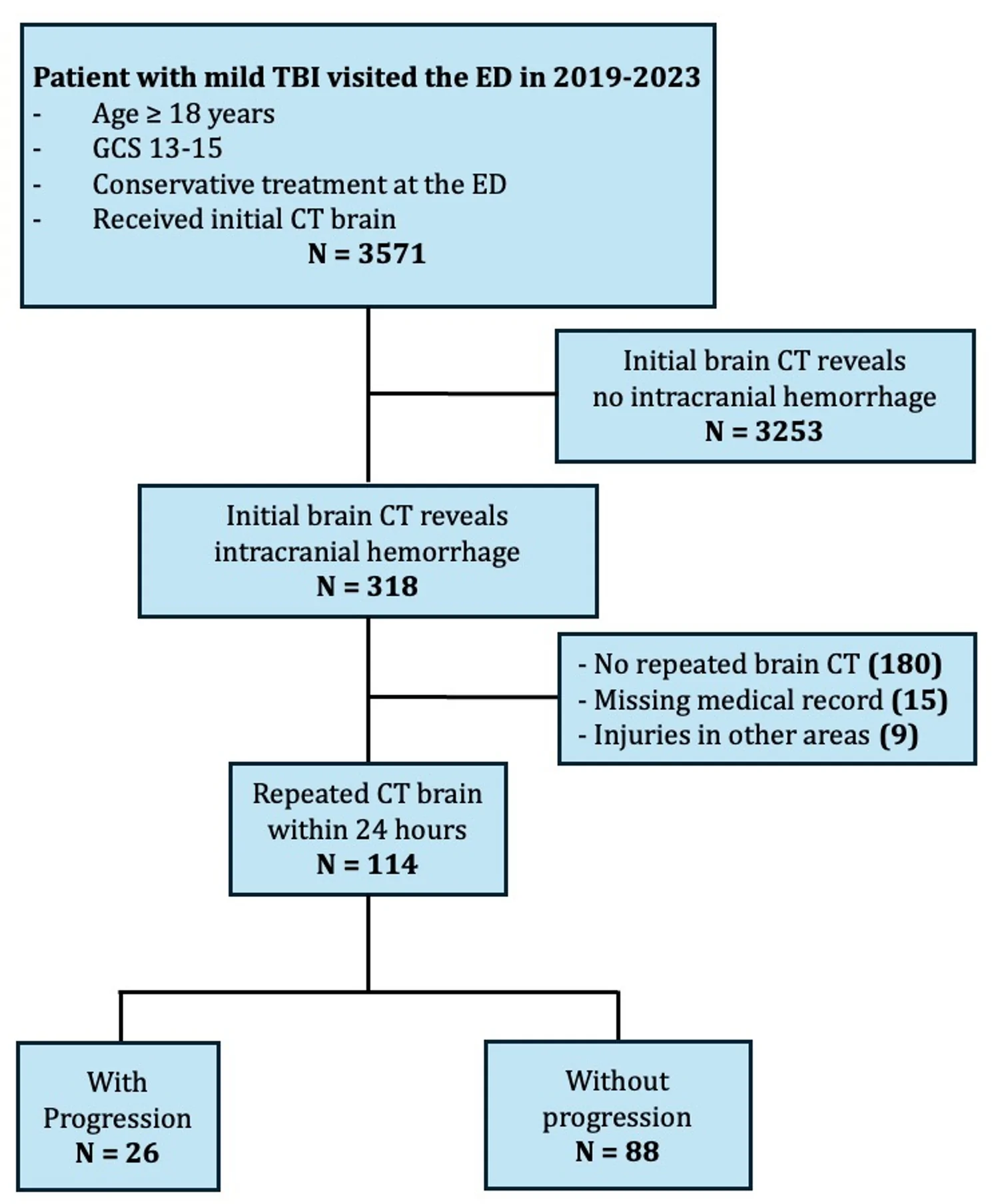
The study flow diagram of patient selection

Among patients with ICH, 180 did not undergo repeat CT imaging. Patients with incomplete clinical or radiological data were excluded from the analysis, including 15 patients with missing medical records and 9 patients with injuries involving other body regions. After applying the inclusion and exclusion criteria, 114 patients who underwent repeat CT brain imaging within 24 h were included in the final analysis. Of these, 26 patients (22.81%) demonstrated clinically significant progression requiring hospital admission or neurosurgical intervention (Fig. 1).

Among patients in the progression group, 16 (61.54%) exhibited clinical deterioration while 25 (96.15%) showed radiologic progression. All patients with progression needed hospital admission, and 12 (46.15%) underwent neurosurgical intervention. In the progression group, the median age of the population was 74 years and the majority were female. The median initial systolic blood pressure was 176 mmHg. The most common mechanism of injury was a fall (84.62%, *N* = 22). Initial GCS 13–14 was observed in 7 patients (26.92%). Hypertension was the most prevalent comorbidity (61.54%, *N* = 16). Some of the patients were receiving antithrombotic therapy, with 10 patients (38.46%) on antiplatelet agents and 11 patients (42.31%) on anticoagulants.

Patients in the progression group more frequently demonstrated multiple ICH lesions (76.92%, *N* = 20), midline shift (46.15%, *N* = 12), and base of skull fracture (7.69%, *N* = 2) on initial CT brain (Table 1).

**Table 1:** Clinical characteristics between mTBI patients with ICH with progression and without progression

Characteristic	With Progression (*N* = 26)	Without Progression (*N* = 88)	*p*-value
Age, median (IQR), years	74 (62, 81)	80 (70, 85.5)	0.024
Male sex, N (%)	11 (42.31)	31 (35.23)	0.644
Onset, median (IQR), hr	2 (1, 3)	2 (1, 6)	0.416
Initial vital signs, median (IQR)			
-Respiratory rate; RR (/min)	20 (20, 22)	20 (20, 20)	0.025
-Oxygen saturation; SpO2 (%)	97 (96, 98)	98 (96, 99)	0.145
-Heart rate; HR (/min)	76 (68, 88)	79 (70, 87)	0.754
-Systolic blood pressure; SBP (mmHg)	176 (154, 193)	149 (129, 167)	<0.001
-Glasgow coma scale; GCS, N (%)			0.041
-GCS 13-14	7 (26.92)	8 (9.09)	
-GCS 15	19 (73.08)	80 (90.91)	
Mechanism of injury, N (%)			
-Traffic injury	2 (7.69)	5 (5.68)	0.658
-Fall from height > 2 meters	2 (7.69)	0 (0)	0.05
-Falling	22 (84.62)	82 (93.18)	0.232
-Others	0 (0)	1 (1.14)	1
Underlying disease, N (%)			
-Diabetes mellitus	7 (26.92)	30 (34.09)	0.635
-Hypertension	16 (61.54)	59 (67.05)	0.642
-Ischemic heart disease	5 (19.23)	22 (25)	0.611
-Cerebrovascular disease	7 (26.92)	26 (29.55)	1
Medication, N (%)			
-Antiplatelet	10 (38.46)	35 (39.77)	1
-Anticoagulant	11 (42.31)	26 (29.55)	0.24
CT scan (CT Brain Non-contrast), N (%)			
-Epidural hematoma	5 (19.23)	7 (7.95)	0.141
-Subdural hematoma	22 (84.62)	68 (77.27)	0.586
-Subarachnoid hemorrhage	18 (69.23)	46 (52.27)	0.177
-Petechial hemorrhage/hemorrhagic contusion	6 (23.08)	7 (7.95)	0.071
-Intraparenchymal hemorrhage	10 (38.46)	7 (7.95)	0.001
-Intraventricular hemorrhage	3 (11.54)	3 (3.41)	0.131
-Skull fracture	10 (38.46)	10 (11.36)	0.003
-Base of skull fracture	2 (7.69)	0 (0)	0.05
-Single ICH	5 (19.23)	52 (59.09)	0.001
-Multiple ICH	20 (76.92)	36 (40.91)	0.002
-Midline shift	12 (46.15)	6 (6.82)	<0.001
Duration from injury to the first brain CT, median (IQR), hours	3 (2, 6)	3 (2, 9)	0.613
Duration from injury to the repeated brain CT, median (IQR), hours	24.5 (15, 30)	25.25 (23.5, 30)	0.317
Outcomes, N (%)			
-Clinical progression	16 (61.54)	12 (13.64)	<0.001
-ICH progression	25 (96.15)	18 (20.45)	<0.001
-Need admission	26 (100)	13 (14.77)	<0.001
-Need neurosurgical treatment	12 (46.15)	1 (1.14)	<0.001
-ED discharge	0 (0)	66 (75)	<0.001
-Revisit ED in 24 hours	0 (0)	0 (0)	1

Abbreviations: ED, emergency department; ICH, intracranial hemorrhage; IQR, Interquartile range

On multivariable analysis, the factors significantly associated with progression of patients with mTBI were midline shift (OR 11.58, 95% CI 2.93–45.78, *p* < 0.001), systolic blood pressure ≥ 160 mmHg (OR 5.49, 95% CI 1.69–17.77, *p* = 0.005), multiple ICH (OR 3.69, 95% CI 1.13–11.98, *p* = 0.030), and initial GCS 13–14 (OR 4.86, 95% CI 1.18–20.08, *p* = 0.029) (Table 2). Receiver operating characteristic (ROC) curve analysis of the final multivariable model demonstrated good discriminatory performance, with an area under the ROC curve (AUC) of 0.878.

**Table 2:** Factors independently associated with progression

Variables	Crude OR (95% CI)	Adjusted OR (95% CI)	*p*-value	AUC
Midline shift	11.71 (3.77–36.34)	11.58 (2.93–45.78)	< 0.001	0.697
SBP ≥ 160 mmHg	3.94 (1.54–10.07)	5.49 (1.69–17.77)	0.005	0.664
Multiple ICH	4.81 (1.76–13.17)	3.69 (1.13–11.98)	0.030	0.680
GCS 13–14	3.68 (1.19–11.42)	4.86 (1.18–20.08)	0.029	0.589

Abbreviations: SBP, systolic blood pressure; ICH, intracranial hemorrhage; CI, confidence interval; AUC, area under the receiver operating characteristic curve

## Discussion

This study found several factors that were independently associated with clinically significant progression in patients with mTBI and ICH, including midline shift, systolic blood pressure ≥ 160 mmHg, the presence of multiple ICH, and initial GCS 13–14. Elevated systolic blood pressure may reflect a physiological stress response rather than a direct causal factor for progression [9].

Initial GCS 13–14 [4], skull fracture [3, 4, 14, 15], multiple ICH [15, 16], and midline shift [17] were identified as significant factors for progression from previous studies, consistent with our findings. Wolf et al. [4] found that GCS < 15 is one of the strongest risk factors used to identify high-risk group, defined as needing neurological intervention or intensive care unit observation, as a lower initial GCS indicates a more critical patient condition. Initial CT findings are also an important factor associated with patient progression. Several studies have shown that patients with skull fracture have an increased risk of ICH, which is associated with worse clinical progression [3, 4, 14]. This is because a skull fracture indicates a higher energy mechanism of injury [18]. A study by Bee et al. [16] reported that patients with multiple lesions in the initial CT group were classified in a worse group, defined as worsening findings on follow-up CT or clinical deterioration requiring intervention, indicating the need for close monitoring. Sumritpradit et al. in 2016 [17] also reported that a midline shift > 2 mm is a factor indicating the need for neurosurgical intervention, as midline shift reflects a mass effect related to hematoma size [19].

In contrast, several studies reported the factors such as anticoagulants [4, 8, 15, 20] and antiplatelet [8, 15, 17] use, SDH [8, 14, 15], EDH [14], and base of skull fracture [16] to be found significantly associated with progression but not found to be statistically significant in our study. The cohort study by Wolf et al. [4] reported that the use of anticoagulants was a significant risk factor in the development of ICH after minor head injury. Kavi et al. [8] study showed that anticoagulant and antiplatelet use are risk factors for poor outcomes, decline in GCS or neurological worsening, and are also associated with isolated SDH, leading to a higher likelihood of requiring surgical intervention. Sumritpradit et al. in 2016 [17] found that patients with clopidogrel use required neurosurgical treatment which shows that it is a significant factor in predicting the progression. The lack of statistical significance in our study may be explained by differences in sample size and statistical power and heterogeneity of antiplatelet exposure (not limited to clopidogrel) compared to other studies. Kavi et al. [8] reported that isolated SDH emerged as a TBI pattern associated with a higher risk of neurological deterioration or the need for neurosurgical intervention. Nevertheless, the underlying mechanism explaining why SDH confers a higher risk compared with other injury patterns remains unclear. Joseph et al. [14] demonstrated that patients with a base deficit ≥ 4 have a substantially increased likelihood of progression on repeat CT brain imaging and exhibit a markedly higher probability of requiring neurosurgical intervention. In our study, only two patients had basilar skull fractures on initial CT, and both showed progression. However, this association did not reach statistical significance, likely due to the small number of patients with basilar skull fractures in our study.

## Limitation

This study has several limitations. First, the retrospective design may have introduced selection bias and limited the completeness and accuracy of the collected data. Some potentially important clinical variables, including loss of consciousness, post-traumatic amnesia, pupillary abnormalities, and focal neurological deficits, were not consistently documented in the medical records and therefore could not be included in the analysis. In addition, repeat CT brain imaging was performed according to physician assessment and institutional practice, which may have introduced selection bias.

Second, the relatively small sample size and limited number of outcome events may have reduced the statistical power of the study, increased the risk of type II error, and limited the stability of the multivariable regression model. This may also have increased the risk of overfitting, as reflected by the relatively wide confidence intervals observed for several predictors. Therefore, the reported associations and discriminatory performance should be interpreted with caution.

Finally, this was a single-center study conducted at a tertiary-care university hospital, which may limit the generalizability of the findings to other clinical settings or institutions.

## Conclusion

These findings may help identify patients at higher risk of clinical and radiological progression among patients with mTBI and ICH. Such findings may assist clinical decision-making regarding monitoring and repeat CT brain imaging. However, further prospective multicenter studies are needed to validate these findings.

## Data Availability

The datasets generated and/or analyzed during the current study are available from the corresponding author on reasonable request.

## Declarations

### Use of artificial intelligence tools

During the preparation of this manuscript, the authors used ChatGPT and Grammarly for language editing and grammar checking. All content was reviewed and edited by the authors, who take full responsibility for the accuracy and integrity of the manuscript.
